# Religion, spirituality, and pediatric mental health: a scoping review of research on religion and spirituality in the Journal of the American Academy of Child and Adolescent Psychiatry from 2000 to 2023

**DOI:** 10.3389/fpsyt.2024.1472629

**Published:** 2024-10-04

**Authors:** Khalid Elzamzamy, Sadiq Naveed, Mary Lynn Dell

**Affiliations:** ^1^ Department of Psychiatry and Behavioral Sciences, Johns Hopkins University School of Medicine, Baltimore, MD, United States; ^2^ Department of Psychiatry, Eastern Connecticut Health Network, Manchester, CT, United States; ^3^ Department of Psychiatry, University of Connecticut, Farmington, CT, United States; ^4^ Frank H. Netter M.D. School of Medicine at Quinnipiac University, North Haven, CT, United States; ^5^ Division of Child and Adolescent Psychiatry, Institute of Living/Hartford Healthcare, Hartford, CT, United States

**Keywords:** religion, spirituality, child and adolescent psychiatry, pediatric mental health, psychiatry and religion, JAACAP, religiosity

## Abstract

**Introduction:**

Religion and spirituality (R/S) serve as sources of meaning-making and coping for many individuals and families. While research on the relationship between R/S and mental health has been ongoing, their role in pediatric mental health is poorly understood. The objective of this study is to assess research trends and predominant themes of R/S in child and adolescent psychiatric research in articles published in the Journal of the American Association of Child and Adolescent Psychiatry (JAACAP). This provides a rough measure of the relative importance of these topics to academic psychiatrists and researchers in the field and identifies gaps for future research.

**Methods:**

All research and review articles published in JAACAP between 2000-2023 with a focus on R/S themes were retrieved, screened, and appraised for content and extent of focus on R/S (major, minor, incidental). Included articles were assessed for R/S variables and predominant themes.

**Results:**

Thirty-two (32) research articles published between 2000-2023 contained sufficient R/S content for inclusion in our study. Only 4 articles had R/S as their major focus. Our analysis suggested a decline in publications with R/S content over the last 24 years. The R/S variables and measurement tools were heterogenous, with religious attendance and religious affiliation being the most frequently measured variables. The predominant themes include the relationships between R/S and psychopathology, suicide, utilization of services, conceptualization of illness, trauma, identity, and coping.

**Discussion:**

Despite the significance attributed to R/S by many youths and families and the increasing academic interest in the intersection between R/S and health, a significant gap exists in our understanding of R/S vis-à-vis child and adolescent mental health. This gap may be further compounded by the limited attention offered to R/S factors and variables in academic psychiatric activities. JAACAP, a global leading academic platform, may advance this discourse by inviting and encouraging publications addressing R/S variables. This may inform diagnostic, preventive, and interventive clinical work with children and their families.

## Introduction

Pediatric mental health researchers and clinicians are consistently searching for ways to better understand and improve the mental well-being of children and adolescents. Despite remarkable progress accomplished over the last few decades with discoveries of new medications and devising new therapeutic interventions, mental health challenges continue to be on the rise, mounting to a national mental health crisis in youth (1). This forces researchers and clinicians to re-examine the dominant areas of focus and work in the field and wonder if there are significant gaps that are less attended to. One such area is the study of religion and spirituality and their relationship to children’s mental health and well-being, as well as their contribution to more holistic forms of assessment and interventions. Increasingly, spirituality is being recognized as a critical determinant of health, influencing not just mental well-being but also broader public health outcomes (2).

As of 2022, about 72% of Americans surveyed by Gallup indicated that religion is very or fairly important in their life. Around 70% indicated that growing up they attended church, synagogue, or mosque every week or almost every week. However, a similar number of Americans indicated that they believe that religion is losing its influence on American life (3). When it comes to youth, according to a 2020 Pew research report, around half of US teens reported attending religious services and having religious or spiritual experiences at least once or twice a month. Moreover, 50-60% reported having participated in a religious education program, such as Sunday school, and having been part of a religious youth group (4).

Religion and spirituality are fundamental sources of meaning, coping, and support for many individuals and families (5). While research on the relationship between religion and physical and mental health has been ongoing for decades (6), the role of religion in the mental health of children and adolescents is an area that has received relatively less attention. While many studies in children and adolescents establish positive associations between religious variables and mental health outcomes, the findings remain far from uniform, with mechanisms of influence and causation links remaining largely unknown. Hence, previous studies have called for further research on the influences of religion across pediatric mental illnesses, settings, and populations (7–9).

Against this backdrop, we hypothesized that there would be increasing interest and numbers of publications in the Journal of the American Academy of Child and Adolescent Psychiatry (JAACAP) that explore the relationship between religious and spiritual variables and mental health outcomes in children and adolescents. In order to test this hypothesis, this study assessed extent, nature, predominant themes, and findings in the study of religion and spirituality in child and adolescent psychiatry in all research papers published in JAACAP between 2000-2023. This provides a rough measure of the relative importance of these topics to researchers in the field and identifies gaps for future research.

Given the prominent role of the Journal of the American Academy of Child and Adolescent Psychiatry (JAACAP) as one of the leading academic platforms for disseminating research in child and adolescent psychiatry globally, this journal serves as a key barometer for academic interest in various topics within the field. The decision to focus exclusively on JAACAP was driven by the journal’s influence in shaping clinical practice and research trends within pediatric psychiatry. By analyzing articles published in this high-impact journal, our study aims to provide insights into how R/S has been addressed—or neglected—within the field over the past two decades. This focus on JAACAP distinguishes our review from previous similar reviews on R/S research in healthcare journals. To our knowledge, all previous reviews were conducted prior to 2000, examined much shorter time frames, and none included a child psychiatric journal, let alone the leading journal of child and adolescent psychiatry. Additionally, unlike earlier studies that may have limited their analysis to specific types of research (e.g., empirical studies), our scoping review includes all research and review articles, providing a comprehensive picture of how R/S has been integrated into the literature (9–18). This approach allows us to identify not only the extent of R/S research but also the thematic and methodological trends within a key publication that significantly influences clinical practice and policy.

### A note on terminology

There is an ongoing academic debate on the definitions of religious and spiritual constructs. In this paper, we use religion and spirituality in the broad sense of the terms to capture all forms of traditional organized religious activities and institutions as well as less institutional forms of spirituality. However, specific papers included in this review may indicate a more specific use of these terms. The abbreviation R/S will be used to denote religion/spirituality and religious/spiritual.

## Methods

### Screening and data extraction

This study utilized a scoping review methodology, chosen because scoping reviews are designed to identify and map the available literature and evidence on a specific topic (19). A scoping review is particularly useful when the literature being examined is heterogeneous in nature. Therefore, this approach was deemed most suitable for our objective of assessing the extent and nature of religion and spirituality research in the Journal of the American Academy of Child and Adolescent Psychiatry (JAACAP) from 2000-2023. The methodology employed follows the five stages presented by Arksey and O’Malley (20).

The data for this study consist of all articles classified as research articles or review articles published in JAACAP between 2000-2023. Given the methodological diversity of papers classified as research articles and review articles in JAACAP, we opted to include all articles under these two classifications, not limiting our analysis to empirical qualitative or quantitative studies. Articles that included R/S themes were extracted through JAACAP’s website by utilizing the following search phrase: “religio* OR spiritu* OR Christian* OR Cathol* OR Protestant* OR Islam* OR Muslim OR Jew* OR Juda* OR Buddhis* OR Hindu* OR church OR mosque OR synagogue OR temple OR pray* OR faith OR existential OR transcenden* OR atheis* OR god OR divine”.

Each identified article was screened for inclusion based on the presence and extent of R/S content. The screening process involved reviewing the abstract, introduction, methods, results, and discussion sections to ensure that R/S was a substantive focus rather than a peripheral mention. Articles that only briefly mentioned R/S in an introductory or discussion sentence or section were excluded.

For articles meeting the inclusion criteria, data extraction involved systematically collecting the following information:

1. Article metadata: Title, authors, year of publication.
2. R/S content: The specific religious/spiritual variables and measures included in the study, if applicable (e.g., religious attendance, affiliation). A religious variable was considered to be any identified religious/spiritual concept or behavior included in data collection and/or data analysis.
3. Focus on R/S: The extent to which R/S was a focus of the study, categorized as major, minor, or incidental as described below.
4. Citation analysis: The number of references cited that included religious or spiritual terms in their titles, to gauge the emphasis on existing R/S literature.
5. Main findings: Key findings related to R/S variables and their association with other variables or outcomes in the study.
6. Methodological approach: The type of study design used (e.g., cross-sectional, longitudinal, review) and how R/S variables were integrated into the research design. Additionally, the study location and sample population were extracted to provide context for the findings. These details are summarized in **Table 1**.

**Table 1:** An overview of all papers included in this scoping review.

Reference	Study design	Sample population (age)	City/Country	R/S Domains	R/S Measures	Study variables	No. of Citations on R/S
Papers with a “major” focus on R/S							
Miller et al. (21)	Cross-sectional	Adolescents (15-19)	USA	Religious affiliation; Belief and practice	Religious denomination; Belief and practice (7 questions)	SUD	9
Miller and Gur (22)	Cross-Sectional	Adolescent girls (mean age: 16)	USA	Personal devotion; personal conservatism; institutional conservatism; participation in religious community	Self-report on seven questions covering the 4 domains.	Physical maturation; Depression	28
Horowitz and Garber (23)	Prospective longitudinal	Adolescents (first assessed in 6th grade) and their parents	USA (Nashville, TN)	Importance of religion and service attendance	Two questions: (1) How often do they go to a place of worship to pray? (2) How important is religion to them?	MDD; IQ; maternal depression	13
Britto (24)	Narrative Review	Arab Muslim Children	USA	Religious/Ethnic Identity Formation	N/A	Arab Muslim identity development	11
Papers with a “minor” focus on R/S							
Cerel et al. (25)	Longitudinal	Children (5-17) and their parents	USA (OH)	Changes in social support and religious practices	Home Environment Interview-Abbreviated Version-Child and Parent Forms (includes changes in religious practices, and religious activities)	Suicide bereavement; psychopathology; family stability; family functioning	0
Offer et al. (26)	longitudinal	Mentally healthy males (14)	USA	Perception of religion as helpful	“Is (Was) Religion helpful to you?”	Autobiographical memory	0
Brook et al. (27)	Prospective Longitudinal Study	Adolescents (12-17)	Colombia	Religious attendance	Single item “How often do you go to church or attend religious services?”.	SUD (marijuana); behavioral problems	0
Drell (28)	Reflections on clinical practice	Hematology/Oncology Patients	USA	General cultural and religious factors	N/A	Psychiatric consultation services	0
Gould et al. (29)	Review	Youth	USA	Religiosity	N/A	Youth Suicide	9
Manne et al. (30)	Longitudinal	Mothers of children undergoing BMT	USA	Religious coping	The COPE inventory (abbreviated 14 items; religious coping)	Maternal coping; depressive symptoms; Pediatric bone marrow transplantation	2
Solomon and Lavi (31)	Cross-sectional	Boys and Girls (11.5–15)	Israel	Religiosity	N/A	political violence and posttraumatic symptoms, future orientation, and attitudes toward peace in	1
Bird et al. (32)	Cross-sectional	Puerto Rican children (3-15)	Puerto Rico & New York City	Intrinsic and extrinsic religiosity	Intrinsic and extrinsic religiosity (3 items): degree of importance; frequency of church/service attendance; religious denomination (33)	Disruptive behavior disorders	1
Bird et al. (34)	Cross-sectional	Puerto Rican children (3-15)	Puerto Rico & New York City	Intrinsic and extrinsic religiosity	Intrinsic and extrinsic religiosity (3 items): degree of importance; frequency of church/service attendance; religious denomination (33)	Disruptive behavior disorders	0
Goodman et al. (35)	Cross-sectional	Schoolchildren (7-14)	Brazil	Religious Affiliation	Religious Affiliation (Catholic, other, mostly Evangelical)	Poor mental health	0
Bird et al. (36)	Longitudinal	Puerto Rican youths	Puerto Rico and New York	Religious denomination; service attendance; importance of religion	A number of scales grouped together to measure family processes. R/S questions adopted from Miller et al. (33)	ADHD; mental health service and medication use	1
Ngo et al. (37)	Description of an evidence-based intervention	N/A	USA	Spiritual coping, such as prayer, meditation, talking to a religious leader, seeking forgiveness, and rituals	N/A	Trauma interventions	0
Morcillo et al. (38)	Longitudinal	Puerto Rican Children (5-13)	Puerto Rico and New York	Religious denomination; service attendance; importance of religion	A number of scales grouped together to measure family processes. R/S questions adopted from Miller et al. (33)	Parental familism; youth antisocial behaviors	1
Wolmer et al. (39)	Controlled Trial	Students in 4th & 5th grades	Israel	Religious affiliation; religiosity; religious school attendance	N/A	Teacher-based preventive intervention; War trauma	0
Han et al. (40)	Cross-sectional	Adolescents (12-17)	USA	Religious activity attendance and beliefs	Two (Y/N) items: 1) Attended religious services in the past year, and 2) my religious beliefs are very important	SUD	2
Mortier et al. (41)	Cross-sectional	First-year college students	8 countries (Australia, Belgium, Germany, Mexico, Northern Ireland, South Africa, Spain, and the United States)	Religious affiliation/background	3-option question (Another religion; No religion; Christian)	Suicidal thoughts and behaviors (STB) in college students	1
Koyanagi et al. (42)	Cross-sectional	Adolescents (12-15)	48 Countries (WHO Data)	Religion-based bullying	Self-report	Bullying victimization; suicide attempts	0
Papers with an “incidental” focus on R/S							
Lyons et al. (43)	Retrospective and cross-sectional	Children in residential placements (5-19)	USA (Florida)	Moral/Spiritual strengths (values, beliefs, service attendance, and church group participation)	Child and Adolescent Strengths Assessment (CASA) (includes “ Moral/Spiritual” strengths domain with 4 items)	Psychopathology in residential settings	0
DuPaul et al. (44)	Cross-Sectional	Preschool-age children (3-5)	USA (PA)	Seeking spiritual support	The Family Crisis Oriented Personal Evaluation Scales (FCOPES) [subscale: seeking Spiritual Support]”	ADHD	0
Ruchkin et al. (45)	Cross-sectional	Juvenile delinquents (14-19)	Russia	Self-transcendence	Temperament and Character Inventory (including “Self-transcendence”)	Posttraumatic stress, psychopathology, violence exposure, and personality traits	0
Hopfer et al. (46)	Review	Adolescent and young adult twins (>11)	USA	Religious affiliation, religious upbringing, and church activity (Koopmans et al., 1999b)	Single item: religious upbringing (no/yes). [Questions measuring affiliation or church activity were not specified]	SUD; environmental and genetic influences of alcohol use initiation	1
Shoal et al. (47)	Longitudinal	Adolescent boys (Starting age: 10-12; Follow-up age: 15-17)	USA (Pittsburgh, PA)	Traditionalism	Multidimensional Personality Questionnaire (MPQ) (“Constraint superfactor” includes “Traditionalism”)	Salivary cortisol concentration; aggressive behaviors	0
Votta and Manion (48)	Cross-sectional	Homeless adolescent males	Canada	Religious coping	COPE inventory (50-items; religious coping)	depressive symptoms, internalizing behaviors, and externalizing behaviors; adjustment; homelessness	0
Lustig et al. (49)	Literature Review	Refugees	USA	Religious figure and healers; Religious beliefs	N/A	Trauma and coping in refugees	0
Yeh et al. (50)	Cross-sectional	Children aged 6-17; Ethnicity: African American, Asian/Pacific Islander American, Latino, or non-Hispanic white (NHW)	Large metropolitan county in the USA	Spiritual etiological factors	Beliefs About the Causes of Child Problems-Parent Version (50) (one category: spiritual causes (10 items)	Etiological explanations of problems	0
Ramrakha et al. (51)	Birth cohort study (prospective longitudinal)	Public health data obtained at ages 5, 7, 9, 11, 13, 15, and 21	New Zealand	Religious activities (participation at Sunday school, attendance at church, church youth group)	Self-report	Childhood problems: Sexual risk taking	0
Merikangas et al. (52)	Cross-sectional	Adolescents (13-18)	USA	Seeking service from a religious/spiritual advisor	Self-report on interview (Utilization of human services including religious advisor)	Mental health service utilization	0
Calvo et al. (53)	RCT	Adolescents with early-onset psychosis	Spain	Family “moral–religious emphasis”	Family Environment Scale (FES) (90-items under 10 categories, one of which is “moral–religious emphasis”)	Psychosis interventions	0

R/S, Religious/Spiritual; SUD, Substance use disorder; MDD, Major Depressive Disorder; IQ, Intelligence Quotient; N/A, not applicable or not available; COPE, Coping Orientation to Problems Experienced; ADHD, Attention-Deficit/Hyperactivity Disorder; WHO, World Health Organization.

### Quantitative analysis

We first identified and counted the total number of articles published in JAACAP between 2000-2023 that met our inclusion criteria. We then calculated the proportion of these articles relative to the total number of research and review articles published during the same period. Additionally, we tracked the distribution of these articles over time to identify trends in the publication of R/S-related content.

### Qualitative analysis

The qualitative analysis involved a scoping thematic review of the included articles. Additionally, to determine the degree to which R/S was an integral part of each article, all articles in the sample were screened and appraised based on the primary topic, the stated objectives, and the extent of data collection and analysis related to R/S. The number of citations concerning R/S was considered a secondary metric, referring to the number of references cited that included religious or spiritual terms in their titles. Each article was then scored on a three-point scale: 1) Major, 2) Minor, and 3) Incidental. This classification approach is similar to that used in previous studies (10). To ensure the reliability and consistency of our scoring, all three authors independently screened and appraised a randomly selected sample of the included papers to resolve discrepancies and reach consensus. The scoring reflects the reviewers’ impressions of the emphasis placed on R/S and the proportion of the text devoted to these concepts, based on the abovementioned primary and secondary metrics. The criteria for each classification are as follows:

1. Major: Articles classified as “major” included R/S terms in their titles and focused primarily on R/S variables in relation to other variables as the study’s primary aim. These articles also typically included a substantial number of citations concerning R/S.
2. Minor: Articles classified as “minor” included some R/S variables among other variables or domains, without particular emphasis on R/S in the study’s aims and objectives. R/S data were collected and included in the analysis and discussion to some extent, with a few citations concerning R/S, if any.
3. Incidental: Articles classified as “incidental” collected some R/S measures, but these were part of a larger inventory that may have included R/S items. These articles did not comment further on these R/S items in their results or discussions, reflecting a low level of intentionality in including R/S, which was also evident in the near absence of R/S citations.

By combining quantitative metrics with qualitative scoping analysis, this study provides a comprehensive assessment of the presence and treatment of R/S topics in JAACAP publications over the last two decades.

## Results

### Quantitative remarks

Between 2000-2023, our search yielded 689 potentially relevant papers classified as research articles or review articles in JAACAP. After reviewing each article to determine if it contained religious/spiritual content, only 120 articles were found to include terms or concepts relevant to R/S. The vast majority of these 120 articles only included a very brief mention of R/S in introductory sentences or while discussing the results. Thirty-two (32) articles were determined as containing sufficient content related to R/S to be included in the study (See **Table 1**. An overview of all papers included in this review). These 32 articles represent 1.13% of the total number (2828) of research and review articles published in JAACAP between 2000-2023. The articles represent a variety of study designs and methodologies. Among them, 15 were cross-sectional studies, nine were longitudinal studies, four were reviews, two were controlled trial (RCT), and two were either descriptive or reflective papers. In terms of geographical representation, the majority of studies were conducted in the United States. The studies covered a range of religious denominations, including Christian, Muslim, and Jewish populations, with many studies exploring religious affiliation more broadly or in multi-religious contexts.

**Figure 1:**
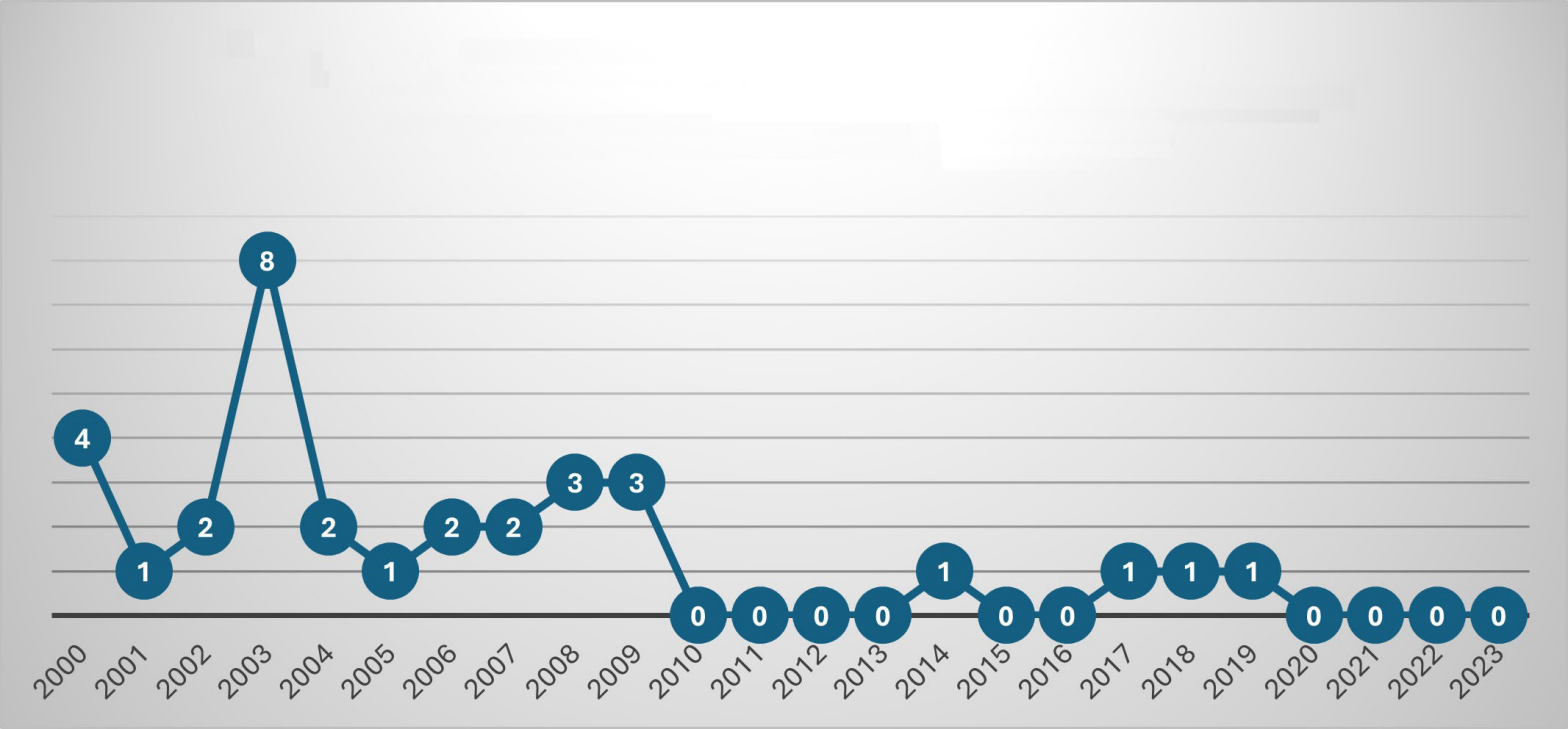
Number of publications with R/S variables or themes per year.

Our analysis showed a declining trend in publications with R/S content. The vast majority of papers in our sample (87.5%) were published between 2000-2009, with the papers that had a major focus on R/S largely concentrated even in the earlier part of this period (2000-2003) (See **Figure 1**. Number of publications with R/S variables or themes per year).

### Religious and spiritual measures

The R/S variables and measurement tools were heterogenous. Eleven studies did not utilize direct measures of religiosity/spirituality but rather used other scales and inventories that happened to include items assessing religious, spiritual, or moral domains. Examples of such measures include the Home Environment Interview-Abbreviated Version (25), the COPE Inventory (30), and the Child and Adolescent Strengths Assessment (43). No studies in our sample used established R/S measures, but rather largely relied on questions used in previous studies on R/S (21, 22), items included in non-R/S inventories (30, 44, 48), or limited-item questions. The most frequently measured R/S variable was religious attendance or participation, followed by religious affiliation and denomination (See **Table 2**. Types and frequency of R/S measures). One study grouped some of those measures under “intrinsic” and extrinsic” religiosity (32, 34), while two other studies conceptualized several of these measures as falling under three broad categories: 1) personal devotion (a personal relationship with the Divine), 2) personal conservatism (a personal commitment to teaching and living according to creed), and 3) institutional conservatism (fundamentalism of religious denomination) (21, 22).

**Table 2:** Types and frequency of R/S measures.

Religious/spiritual domain	Frequency
Religious attendance, activity, and participation	11
Religious affiliation and denomination	8
Religious coping and seeking spiritual support and comfort	7
Importance ascribed to religion	7
Religiosity (uncharacterized)	2
Religious upbringing	1
Centering god when making life decisions	1
Having a religious turning point in life	1
Encouraging others to believe in one’s religion/g-d	1
Believing one’s scriptures to be the actual word of g-d, without mistakes, and that has to be taken literally	2
Self-transcendence (comfort in spiritual experiences and beliefs in supernatural power)	1
Expression of religious beliefs	1
Traditionalism (linked to high moral standards and support of religious values)	1
Changes in religious practice	1
Spiritual etiological factors of diseases	1
Family religious and moral emphasis	1
Attendance of religious schools	1
General religious factors	1
Religion-based bullying	1
Religious identity	1
Perception of religion as helpful	1
Being a “born-again” Christian	1
Relative fundamentalism of religious denomination	1

### Degree of focus on R/S

Only four articles, representing 12.5% of our sample and 0.14% of the total number of research and review articles published between 2000-2023, were determined to have a “major” focus on R/S as indicated by the inclusion of R/S constructs in their titles and objectives, as well as the extensive citation of previous R/S literature (21–24). Three of these articles were empirical in nature, while one was a narrative review (24). A slight majority of our sample (53.1%, n=17) were determined to have a “minor” focus on R/S, while 34.4% (n=11) were classified as “incidental”. Overall, only 13 articles (40.6% of our sample) included citations to previous R/S literature.

### Qualitative findings

Our thematic review of the papers revealed a wide range of contexts and associations within which R/S were examined. These include associations with psychopathology, suicide, trauma-related symptoms and response, coping, identity, utilization of services, and explanatory models of illnesses. See **Table 3** for summary of findings.

**Table 3:** Summary of findings.

Significant Associations or Findings	
There was an inverse relationship between earlier adolescent marijuana use and later religious attendance	Brook et al. (27)
The magnitude of genetic influence on substance use is moderated by a range of factors including religiousness (more religious, less genetic influence)	Hopfer et al. (46)
Personal devotion and institutional conservatism were inversely associated with the use of contraband drugs, alcohol consumption (+ personal conservatism), substance dependence, and abuse, in a largely white Christian sample.	Miller et al. (21)
Religiosity’s protective effects against depression varied by physical maturation status in adolescent girls. Personal devotion and frequent religious service attendance were associated with a significant decrease in depression among highly mature adolescent girls. By contrast, personal devotion and institutional conservatism were inversely associated with depression in non–highly mature girls	Miller and Gur (22)
Depressive episodes during grades 7 through 11 predicted lower religious attendance during 12th grade, controlling for prior religiosity.	Horowitz and Garber (23)
Religious attendance in 6th grade predicted lower odds of developing depressive diagnoses during grades 7 through 12 (marginal significance)	Horowitz and Garber (23)
A very high percentage of religious Jewish youth was found in the participants residing in the Jewish settlements in disputed territories compared with other regions (e.g., Jerusalem and Gilo)	Solomon and Lavi (31)
A statistically significant increase in self-transcendence scores (reflects finding comfort in spiritual experiences and beliefs in supernatural power) was found in participants with full PTSD compared to participants with no or partial PTSD.	Ruchkin et al. (45)
The second strongest correlate of lifetime suicidal thoughts and behaviors was having a religion other than Christianity	Mortier et al. (41)
Religion-based bullying was found to confer the highest odds for suicide attempts.	Koyanagi et al. (42)
Spiritual etiological causes were among the three least often endorsed causes among four racial/ethnic groups.	Yeh et al. (50)
Rates of lifetime utilization of human services (including religious advisors) by adolescents with any class of DSM-IV disorder was lower than the utilization of mental health specialty services and school services.	Merikangas et al. (52)
Religious coping strategies were commonly used by mothers of children undergoing bone marrow transplantation.	Manne et al. (30)
Participation in church youth groups was among the least common strengths reported by children and adolescents in residential settings.	Lyons et al. (43)
No Significant Associations or Findings	
A declining trend in religious activity attendance and beliefs from 2002 through 2014 was identified. However, this declining trend was not correlated with the downward trend in substance use among youth.	Han et al. (40)
No association was found between substance abuse and a rigid adherence to creed (personal conservatism)	Miller et al. (21)
Religiosity was not found to moderate the relation between chronicity of maternal depression and depressive disorders in adolescents (i.e., the protective effects of religiosity were not strong enough to counter the effects of maternal depression on depressive episodes).	Horowitz and Garber (23)
No significant association was found between religiosity and disruptive behavior disorders in Puerto Rican children.	Bird et al. (32, 34, 36)
No significant difference in “seeking spiritual support” between preschool children with ADHD and controls.	DuPaul et al. (44)
No significant associations between low salivary cortisol concentration, aggressive behavior, and Traditionalism (related to moral and religious values).	Shoal et al. (47)
No significant association between religious affiliation, as a potential risk factor, and poor child mental health.	Goodman et al. (35)
Religiosity was not found to be contributory to the relationship between behavioral and emotional problems during childhood and later sexual risk-taking in young adulthood	Ramrakha et al. (51)
No effect for religious variables on symptoms of post-trauma and stress/mood in children exposed to rocket attacks in Israel.	Wolmer et al. (39)
There were minimal changes in religious practices of children following parental death by suicide, with no significant differences between suicide-bereaved and non-suicide-bereaved children.	Cerel et al. (25)
No difference observed in the two study groups in the perception of the family’s “moral-religious emphasis” pre- and post- treatment for early onset psychosis.	Calvo et al. (53)
Accuracy of autobiographical memory for religious and most other items tested was no different.	Offer et al. (26)
Religious coping was not significantly associated with depressive symptoms, internalizing behaviors, or externalizing behaviors in homeless youth.	Votta and Manion (48)
Other Findings	
Spiritual coping was endorsed as part of a school-based trauma intervention (Cognitive-Behavioral Intervention for Trauma in Schools, CBITS)	Ngo et al. (37)
Reaction to stress in refugee populations was seen as possibly mediated by belief systems.	Lustig et al. (49)
Religiosity was endorsed as a potential protective factor against suicide.	Gould et al. (29)
The consideration of cultural and religious factors was endorsed when working with patients and their families receiving hematology/oncology services.	Drell (28)
A review endorsed the exploration of how a Muslim identity contributes to the identity development of Arab Muslim children in the USA.	Britto (24)

#### Religion and psychopathology

These studies explore how R/S influences mental health outcomes across a range of disorders, including substance use disorders, depressive disorders, attention-deficit/hyperactivity and impulse control disorders, trauma and stress-related disorders, and suicidal behavior. Additionally, research has examined how spiritual beliefs contribute to the understanding (etiology) and treatment of these psychopathologies. The findings are diverse, reflecting the complex roles that R/S can play in mental health, with some studies highlighting protective effects while others show minimal or no significant associations. Below, we review the key findings within specific psychopathological domains, as well as the role of spiritual explanations and treatments for these conditions.

##### Substance use disorders

The relationship between substance use and religiosity was featured in a few papers. Brook et al. (27) examined the relationship between earlier adolescent marijuana use and later adolescent behavioral problems in a community-based sample of Colombian youth. The results showed that earlier adolescent marijuana use had an inverse relationship with later religious attendance. Hopfer et al. (46) reviewed twin and adoption studies of adolescent substance use to explore the impact of the family environment, including religiosity, on genetic expression. One of the studies included in this review indicated that the magnitude of genetic influence on substance use is moderated by a range of factors including religiousness (more religious, less genetic influence). A large data study was conducted by Han et al. (40) examining the trends in the 12-month prevalence and patterns of substance use among US youth from 2002 through 2014. The study identified declining trends in religious activity attendance and beliefs from 2002 through 2014. However, this declining trend was not correlated with the downward trend in substance use among youth.

To replicate previous findings from studies on adult substance use that showed an inverse association between religiosity and substance use, Miller et al. (21) focused specifically on examining the protective effects of religiosity against substance use and abuse in a nationally representative sample of adolescents. The main finding in this analysis was that personal devotion and institutional conservatism, but not personal conservatism, were inversely associated with the use of contraband drugs, alcohol consumption (+ personal conservatism), substance dependence, and abuse. Authors inferred that “no association was found between substance abuse and a rigid adherence to creed.” (p. 1197). The authors engaged in an extensive analysis of these findings, comparing them with the results in adult samples, and examining them in light of religious development notions. A limitation of this cross-sectional study was the predominantly white Christian sample which cannot be generalized to other religious groups (21).

##### Depressive disorders

The relationship between depression and religiosity was the focus of two studies from different perspectives. Horowitz and Garber (23) prospectively examined the relation of IQ and religiosity to depressive disorders in adolescents with a maternal history of depression (with respect to the chronicity of their mothers’ depression history). They found that IQ, but not religiosity, moderated the relation between chronicity of maternal depression and depressive disorders in adolescents. In other words, the potential benefits of religiosity were not found to buffer the effects of maternal depression on depressive episodes. In other words, the potential benefits of religion were not enough to counter the effects of maternal depression on children. Additionally, “depressive episodes during grades 7 through 11 predicted lower religious attendance during 12th grade, controlling for prior religiosity. There also was a marginally significant trend for religious attendance in 6th grade to predict lower odds of developing depressive diagnoses during grades 7 through 12.” (p. 578). Overall, the authors concluded a possible bidirectional relation between religious attendance and adolescent depression. Of note, the authors offered in-depth introductory and discussion remarks on studying and conceptualizing the relation between religiosity and psychopathology in children (23).

Miller and Gur (22) on the other hand, assessed the impact of physical maturation on the protective qualities of religiosity against depression in adolescent girls. Within the full sample, personal devotion, frequent participation in a religious community, and institutional conservatism were inversely associated with depression, while personal conservatism was not significantly associated. In terms of the main outcome of the study, the association between religiosity and depression differed by physical maturation status in this nationally representative sample of adolescent girls. Personal devotion and frequent attendance of religious services were associated with a 50% to 200% greater decrease in the likelihood of depression in highly mature girls than in non–highly mature girls. By contrast, personal conservatism and institutional conservatism were inversely associated with depression in non–highly mature girls, but not in highly mature girls. The authors discussed potential mechanisms underlying the differential associations between religiosity and depression by maturation status (22).

##### Attention-deficit/hyperactivity and impulse control disorders

Religious/Spiritual variables, among other factors, were assessed in relation to Attention-Deficit/Hyperactivity Disorder (ADHD), disruptive, or aggressive behaviors. In a sample of Puerto Rican children, religiosity was not significantly associated with disruptive behavior disorders (32, 34). In kids with ADHD, differences in home, school, and medical functioning between preschool-age children with ADHD and normal control children were examined. No significant difference was found in “seeking spiritual support”, although total raw scores on the FCOPES scale indicated statistically significant greater family dysfunction in the ADHD group (44). Shoal et al. (47) tested the relationship between low salivary cortisol concentration in preadolescent boys and aggressive behavior later in adolescence, and whether personality traits mediate this relation. The results indicate no significant associations with Traditionalism (related to high moral standards and religious values). A study found no significant association between religious affiliation, as a potential risk factor, and poor child mental health (35). Finally, a birth cohort study examining behavioral and emotional problems during childhood that may predict sexual risk-taking in young adulthood adjusted for religiosity and found no significant contribution of religiosity to the results (51).

##### Trauma and stress-related disorders

Religious themes appeared in several studies examining trauma-related symptoms and interventions.

Two studies out of Israel considered religious variables in their data and/or analyses. Wolmer et al. (39) studied the effects of a teacher-based preventive trauma intervention implemented with Israeli fourth and fifth grade students in a city in southern Israel who were exposed to continuous rocket attacks during Operation Cast Lead in 2008. The results showed no effects for religious variables on symptoms of post-trauma and stress/mood. The percentage of children meeting the cut-off criteria for PTSD was similar in religious and nonreligious schools in the intervention and the control groups (39). An earlier study by Solomon and Lavi (31) examined the relationship between exposure to political violence and posttraumatic symptoms, future orientation, and attitudes toward peace in a sample of boys and girls from Jerusalem, Gilo, and the Jewish settlements in the disputed territories. The study reported a very high percentage of religious youths (93.1%) in the participants residing in the disputed territories compared with Jerusalem (0%) and Gilo (one participant). The authors attempted to explain one of the results of the study in light of this difference in religiosity stating “The most optimistic youths were the ones from the disputed territories who had experienced the most intensive terror and who reported the highest level of PTSD symptomatology. This surprising finding may be partly explained by the strong religious beliefs and ideological convictions of these adolescents.” (p. 1173). The authors here alluded to the role that religion could play in meaning-making following traumas and tragedies. No further analysis of religious variables in relation to other outcomes was attempted in this study (31).

One paper illustrated an outline for a trauma intervention entitled Cognitive-Behavioral Intervention for Trauma in Schools (CBITS). CBTIS is an evidence-based program developed to meet the needs of underrepresented ethnic groups and immigrant youths exposed to trauma. One of the five main treatment components is “social problem solving”. The component blends in aspects of spiritual coping, such as prayer, meditation, talking to a religious leader, seeking forgiveness, and rituals, as potential actions to encourage in faith-based schools and with diverse groups of students where this is an important part of their culture/context (37).

Traumas and stressors related to the refugee populations and the role of religion in coping were discussed in a literature review paper examining child and adolescent refugee mental health. Religion was featured a few times in the paper from the lens of facilitating coping. The overarching conclusion from this study was that reactions to stress may be mediated by coping strategies, belief systems, and social relations (49).

Post-traumatic stress was studied in relation to comorbid psychopathology, violence exposure, and personality traits in a sample of Russian juvenile delinquents. The study found a statistically significant increase in self-transcendence scores (reflects finding comfort in spiritual experiences and beliefs in supernatural power) from the no PTSD, to the partial PTSD, to the full PTSD group. However, the author failed to interpret or comment on this finding in their discussion of the results while they attempted to interpret the other temperament and character-related findings (45).

##### Suicide-related themes

Cerel et al. (25), in a comparative study, examined suicide-bereaved (SB) children after parental death. One of the primary hypotheses was that religious practices after deaths by suicide are typically influenced by religious stigma resulting in social withdrawal, disconnectedness, and guilt. The results showed minimal difference (increase or decrease) in religious practices between groups (25). Another study examined the lifetime prevalence and correlates of suicidal thoughts and behaviors (STB) among a large sample of first-year college students from 8 countries. The strongest correlate of lifetime STB was sexual orientation followed by having a religion other than Christianity. Sexual orientation was also the strongest correlate of transitioning from ideation to plan, followed by having a religion other than Christianity (41). Another religious variable, namely religion-based bullying, was found to confer the highest odds for suicide attempts followed by bullying due to other reasons such as race, in a large international study assessing the association between bullying victimization and suicide attempts (42). Finally, in a critical review of 10 years of research on youth suicide, Gould et al. (29) identified two main themes of protective factors namely religiosity and family cohesion. The authors identified works that documented the protective role of religiosity against suicidal behavior in adolescents and young adults. They also identified potential limitations to those studies such as not controlling for potential confounders. The studies included in this review were mostly published before 2000 and none of them were published in JAACP (29).

##### Spiritual explanations of psychopathology

Yeh et al. (50) examined racial/ethnic patterns of parental beliefs about etiological explanations of children’s problems. Spiritual etiological causes were among the three least often endorsed causes among all four racial/ethnic groups. There were no significant racial/ethnic differences for “spiritual issues”, while there were significant racial/ethnic differences in 7 (out of 11) other etiological categories.

##### Spiritual treatments for psychopathology

In a nationally representative sample of youth, rates and sociodemographic correlates of lifetime mental health service use were examined. Respondents were asked whether they had ever received services for emotional or behavioral problems and the settings in which they had received these services. Although the data included a “human services” category, which includes services offered by a “counselor, a religious/spiritual advisor, or mental health crisis hotlines”. The study did not provide separate figures for religious/spiritual advisors versus other human services. Rates of lifetime utilization of human services by adolescents with any class of DSM-IV disorder was 18.2%, compared to utilization of mental health specialty services (46.5%) and school services (35.4%) (52).

#### Family’s religious environment

Several studies examined certain religious factors in the family environment and family system in relation to psychopathology or interventions.

In a randomized controlled trial, Calvo et al. (53) assessed the efficacy of a structured psychoeducational group intervention for adolescents with early-onset psychosis and their families. Although religiosity was not a primary focus of the study, a family environment measure included an item on “moral–religious emphasis”. There was no difference observed in the two study groups in the perception of the family’s “moral-religious emphasis” pre- and post-treatment. Another study by Morcillo et al. (38) examined the relationship between parental familism (strong values of attachment to nuclear and extended family members) and youth antisocial behaviors (ASB) over time. Religiosity was measured as part of a scale measuring family processes borrowed from an article published in JAACAP in 1997. The finding, although did not stratify religious versus other “family influences” factors indicated that “family influences” were a potential mediator of the relation between parental familism and ASB in young boys only. The study found that parental familism was inversely related to ASB over time. Similarly, negative family influences, subsuming religiosity, were inversely related to the likelihood of services use and medication use in Puerto Rican youth (36).

A longitudinal study by Offer et al. (26) examined the accuracy of autobiographical memory in more than 60 subjects who were studied in 1962 at the age of 14 and then interviewed face to face at the age of 48. Questions concerning various areas of life included a question on religion “Is (Was) Religion helpful to you?”. Subjects’ memories at the age of 48 were no better than chance when remembering whether they had stated as adolescents that religion was helpful. The same was also true for the majority of items asked about.

#### Religious coping, identity, and strengths

Two papers engaged with religious themes in relation to coping and physical health problems. In a “clinical perspectives” article by Drell (28) highlighting lessons learned in the process of consulting at a weekly hematology/oncology psychosocial conference, the author dedicated a section to explore cultural and religious factors worth taking into consideration when working with patients and their families with specific case vignettes. On the other hand, Manne et al. (30) evaluated the role of maternal coping strategies in depressive symptoms experienced by mothers of children undergoing bone marrow transplantation (BMT). The study found that religious coping was among the most commonly used strategies by mothers. The use of religion was associated with the course of depressive symptoms, but the magnitude of the associations differed depending on the use of the coping strategy at the time of transplantation. The authors highlighted a unique aspect of this study on religious coping which examined the potential interaction effects of religious coping with the time and degree of its use. Another study examining coping and adjustment in homeless youth found that “engagement coping styles”, including religious coping, were not significantly associated with the dependent variables (depressive symptoms, internalizing behaviors, and externalizing behaviors) (48).

Children and adolescents in residential settings were assessed from a strength-based approach by Lyons et al. (43). Overall, there was substantial variation across individuals and the types of strengths they identified with. Participation in church youth groups was among the least common strengths reported. The authors hypothesized that the placement in a residential setting could have limited the ability of some participants to attend church groups, although it did not eliminate this opportunity for all participants. Finally, higher levels of almost all strengths, including moral/spiritual strengths, were associated with lower symptoms, risks, and functional impairment (43).

Finally, A review paper by Britto (24) explored the factors influencing the identity development of Arab Muslim children in the US by surveying the multidisciplinary body of research examining issues linked to the topic. After presenting a brief historical overview of Arab Muslim immigration to the United States, the author expanded on the literature on Arab Muslim Children and Adolescents which was conceptualized as divided into three broad areas: research on Muslims, research on Arabs, and research on Arab Muslims. Finally, the author recommended further research to develop a cohesive conceptual framework that would fully explicate the ethnic identity of Arab Muslim children which is characterized by an interplay of multiple unique dimensions (24).

## Discussion

This review of the published research concerning R/S in child and adolescent psychiatry revealed several findings. The aggregate figure of the total number of articles with content related to R/S (1.35%) is lower than that found in previous studies examining R/S research in various healthcare-related fields and journals and in much shorter time frames, such as adolescent research journals (11.8%; years 1992 to 1996), American Psychological Association journals (2.7%; years 1991-1994), mental health nursing journals (10%; years 1991-1995), Journal of Traumatic Stress (4.7%; years 1990–1999), nursing research journals (13.5%; years 1995–1999); palliative care journals (18.5%; 1990-1999), marriage and family journals (13.2%; years 1995-1999), and gerontology journals (3.7%; years 1985-1991) (9–16). However, the figure is comparable to the findings of a similar study that examined R/S in four major psychiatric journals between 1991-1995 (1.2%), yet still lower than the finding in the same journals between 1978-1982 (2.5%) (17, 18). This indicates suboptimal attention paid by pediatric psychiatry researchers to the role of religious factors in pediatric mental health, despite a plethora of indicators of the significance of R/S to pediatric mental well-being and psychopathology. This deficit is further demonstrated by the limited use and citation of available literature on R/S and mental health where less than half of our sample of articles on R/S included such citations as shown in **Table 1**.

Our initial hypothesis posited that there would be an increasing interest and number of publications in JAACAP exploring the relationship between R/S and mental health outcomes in children and adolescents over the study period from 2000 to 2023. However, the findings of our review did not support this hypothesis. Instead, the data revealed a relatively low and even declining trend in the number of publications addressing R/S topics within JAACAP during the specified timeframe.

As far as the degree and extent of focus on R/S, our study revealed that the vast majority of papers initially extracted included only passing brief mentions of R/S. Additionally, the majority of articles with more elaborate engagement with R/S only did so to a minor extent or even trivially and incidentally, leaving only four articles with a major focus on R/S, three of which were empirical in nature. That being said, our study indicates an improvement from a previous study that examined four major psychiatric journals for religious variables between 1991-1995. That study found almost no psychiatric research that used a religious measure to appraise an adolescent population (18).

Measurement of R/S requires a thoughtful process. R/S are multi-dimensional experiences with intra-psychic, behavioral, interpersonal, social, and supernatural elements. In our pediatric population, this multidimensionality is further compounded by significant developmental and familial elements. Moreover, the relationship between R/S and mental health is a complex one, requiring the development of measures that reliably and accurately assess R/S. Frequently used narrow measures, such as frequency of service attendance or religious affiliation (the most widely used measures in our sample), although offer some data, do not account for the richness and complexity of the R/S experience and do not capture the mechanism through which R/S can affect health and health outcomes (54–56). Some of the measures utilized in our sample were rooted in Christian-based beliefs but represented a somewhat multidimensional approach examining a range of values and behaviors (21, 22).

What potentially contributed to the limitation of R/S measures in a portion of our sample is the reliance on national adolescent health databases which incorporate only one or two R/S variables, if any, typically limited to religious affiliation and/or religious service attendance (57). Several R/S measures were used in previous studies for adolescent health outcomes research and show promise such as 1) the Brief Multidimensional Measure of Religiousness/Spirituality, 2) the Spiritual Well-Being Scale, 3) RCOPE, and 4) the Religious Orientation Scale (8, 57). These measures have been designed for adults but have been used in adolescent research. Some efforts to develop R/S measures specifically relevant to adolescents include the Religious Behavior Questionnaire (58) and the Religious Attitudes and Practices Survey (59).

Our analysis also demonstrated a plethora of what could be described as missed opportunities for including and/or analyzing religious/spiritual variables. Whether intentional or not, a number of studies used scales or datasets that included some R/S items, yet, failed to analyze and/or interpret those variables in relation to their outcomes (60–68). Some otherwise well-designed studies in our sample could have been strengthened by including and analyzing religious/spiritual variables. For example, there have been a large number of studies that indicated in their introductions or discussions that R/S could have offered an explanatory or helpful measure but was beyond the scope of their studies (69–74). Another example of a missed opportunity is the large number of prospective studies (n=10, 31%) in our sample which could have offered opportunities for establishing causation links to R/S. Experts on R/S and health research identified a dire need for more longitudinal studies on the topic (75). Some studies explicitly excluded religious/spiritual dimensions despite being part of the inventory or relevant to the target problem being examined (76–79). This lack of inclusion and assessment of R/S occurred despite a plethora of literature establishing associations between R/S and the themes under study.

In terms of the descriptive finding and predominant themes in the literature, a few observations can be made. Various religious domains had overall inverse relationships with substance use variables, including moderating genetic influences on substance use. This is in accord with previous research that provided evidence that various religious factors may confer protection against substance use problems in adolescence, with one review finding that it is the strongest protective association among all psychopathologies (8, 80).

A similar protective association was noted against depression (22); however, it differed by physical maturation status. In contrast, religiosity was not found to provide a potentially protective effect against depression in adolescents with mothers suffering from depression. There was an interesting bidirectional influence between religious attendance and adolescent depression, where depressive episodes predicted lower religious attendance while higher religious attendance seemed to confer some protection against depression (23). Existing literature on the relationship between depression and R/S in adolescents revealed a nuanced relationship between certain religious variables and depressive symptoms. Some variables such as positive interpersonal religious experiences and religious meaning-making were found to be associated with less depressive symptoms, while religious struggles and negative interpersonal religious experiences were associated with more depressive symptoms (7, 81). As Dew et al. (8) noted, the exact relationship between adolescent depression and religious variables remains ambiguous, as more longitudinal studies are needed.

While the overall protective role of religion against suicide was emphasized in some of the papers in our sample, religion as an identity was the target of bullying leading to a higher risk for suicide attempts. The finding that a religion other than Christianity was strongly correlated with lifetime suicidal thoughts and behaviors is difficult to interpret given that more nuanced multi-dimensional measures of R/S were not utilized. The majority of prior research indicated that religiosity seems to be associated with lower levels of suicidality, however, a potential bidirectional relationship should not be undermined (8).

Interesting findings on the connection between R/S and trauma-related responses were reported. A finding of increased self-transcendence scores in children with PTSD compared to no PTSD, although not interpreted by the authors, could indicate an element of “post-traumatic growth” where individuals attempt to find comfort in connecting to a higher power in response to tragedies and traumas. This is potentially corroborated by the finding of a higher percentage of religious youths in war-zone areas (31, 82) as well as the high utilization of religious coping in mothers of medically ill children (30). Therefore, including religious coping in trauma-focused interventions is an appropriate and much-welcome addition as was seen in Ngo et al. (37). Some emerging literature has emphasized the need to explore modes of post-trauma resilience, and the role played by R/S in such. Various patterns of religious/spiritual coping, some positive and some negative, have been described and studied (83). Among children and adolescents, religion was found to be associated with resilience after trauma (84).

Although the studies in our samples explored a variety of themes in relation to R/S, more contributions are needed to inform the growing interest and significant gap in the literature on the relationship between R/S and mental health outcomes. Although existing research indicates certain general trends in the relationship, there are yet tremendous dimensions to uncover: which religious variables relate to which internalizing or externalizing symptoms? Which measures work well for measuring what? Which religious/spiritual variables confer risk to mental health outcomes? Which R/S variables contribute to help-seeking or act as barriers? Those questions can have significant value for research and clinical purposes. For example, the finding that R/S variables consistently strongly correlate with substance use outcomes led some researchers to recommend controlling for substance use when studying religion’s relationship to any type of mental health outcome. That is, “Protection from substance use may serve as an important mechanism through which religion acts to promote all types of mental and possibly physical health.” [(8), p. 393].

## Conclusion and future directions

Despite the increasing global academic interest in the intersection between R/S and health outcomes, child and adolescent mental health literature on the topic seems to continue to suffer significant gaps. Our initial hypothesis proposed that there would be an increasing interest in and number of publications within JAACAP that explore the relationship between R/S and mental health outcomes in children and adolescents from 2000 to 2023. However, our findings did not support this hypothesis. Instead, the data revealed a relatively low and declining trend in the number of publications addressing R/S topics within JAACAP over the study period. Furthermore, the degree of focus on R/S was generally limited, with most articles offering only brief mentions or minor engagement with R/S themes. Only a small fraction of the studies had a major focus on R/S. In light of this, it is increasingly important to recognize spirituality not only as a personal or cultural element but also as a significant determinant of health that can shape mental health outcomes and inform public health strategies (2).

JAACAP, being one of the leading child and adolescent psychiatry journals globally is well positioned to advance this discourse by inviting and encouraging submissions that address R/S variables. This can be done through special issues on R/S but also through emphasizing and encouraging the inclusion of R/S variables in empirical studies. Bridging this gap is not only critical for research and academic purposes, but it can also inform diagnostic, preventive, and interventive clinical work. AACAP acknowledges the significant role played by religion in youth mental health and development and the potential for religious distress and conflicts resulting in situations requiring clinical and/or spiritual interventions (85). A large number of studies published in JAACAP also proposed a potential role played by R/S in influencing their outcomes, however, only a small number of them took on the task of measuring R/S. As we hope more researchers in the future will consider measuring R/S constructs and their relation to pediatric mental health outcomes, it is necessary to realize the need for measuring constructs that are possibly theoretically and functionally linked to those outcomes and are relevant to the target population. In addition, future research needs to consider using validated multidimensional measures and measures that capture and address the diversity and heterogeneity of children and youth in their samples.

It is the hope of the authors that including our children’s and adolescents’ R/S experiences would provide psychiatrists and mental health clinicians with a better understanding of their overall health and well-being. In conclusion of this analysis, one cannot help but wonder: if this is the status of the inquisitive, research side of our field over the last 22 years in relation to R/S, how are we doing in the practical, clinical side of things and how much attention are we are paying to R/S themes in our assessments and interventions, which is an area of a growing need for training and capacity building?

## Data Availability

The original contributions presented in the study are included in the article/supplementary material. Further inquiries can be directed to the corresponding author.
